# The influence of conventional and novel blanching methods on potato granules, phytochemicals, and thermal properties of colored varieties

**DOI:** 10.3389/fnut.2023.1178797

**Published:** 2023-05-05

**Authors:** Rajni Saini, Sukhpreet Kaur, Poonam Aggarwal, Atul Dhiman

**Affiliations:** ^1^Department of Food Science and Technology, College of Agriculture, Punjab Agricultural University, Ludhiana, Punjab, India; ^2^Department of Food Science and Technology, Dr. Yashwant Singh Parmar University of Horticulture and Forestry, Solan, Himachal Pradesh, India

**Keywords:** phytochemicals, starch, colored potato, blanching methods, functional group

## Abstract

**Introduction:**

Colored potatoes comprise many bioactive compounds that potentially support human health. Polyphenols present in them have associated therapeutic benefits like antimutagenic and anticarcinogenic properties.

**Method:**

The current study aimed to explore the effects of different blanching methods (steam blanching, hot water blanching, and microwave-assisted blanching) on the phytochemical and structural aspects of *PP-1901* and *Lady Rosetta* (LR) potato varieties. Changes in the antioxidant activity, color, total ascorbic acid, phenolic, and flavonoid content were based on the variations in parameters including temperature (blanching using hot water and steam) and capacity 100– 900 W (blanching using microwave).

**Results:**

For both *PP-1901* and LR varieties, all the blanching methods led to a significant reduction in residual peroxidase activity, as well as affecting their color. The preservation of bioactive substances exhibited a microwave steam>hot water blanching trend. Blanching significantly increased the antioxidant activity of all the samples. Additionally, Fourier-transform infrared spectroscopy revealed that phytocompounds were retained to their maximum in microwave-blanched samples, especially at 300 W. The type of blanching method significantly affected the thermal properties of potatoes by disrupting the ordered structure of the matrix.

**Discussion:**

Microwaves at 300 W can be used as a novel and suitable alternative technique for blanching potatoes, which successfully retained the original quality of it in comparison to steam and hot water blanching.

## 1. Introduction

Potatoes are the fourth most important crop for human consumption on a global scale after maize, rice, and wheat (1). Potatoes are rich sources of micronutrients like vitamin B_1_ (106 mg/g), vitamin B_3_ (400mg/g), vitamin C (130 mg/g), and iron (0.31 mg/g), as well as minerals like potassium (379 mg/g), phosphorus (44 mg/g), calcium (5 mg/g), and magnesium on a dry basis. They also contain niacin, folate, and riboflavin, as well as various phenolic compounds which increase the antioxidant capacity of potatoes (2, 3). Potato polyphenols have therapeutic effects associated with them such as anticarcinogenic and antimutagenic activities (4). Colored potatoes have up to eight times higher antioxidant potential compared with their white or yellow counterparts (5). Research has found that colored potatoes are a healthier choice for consumers as they possess greater levels of bioactive compounds and anticancer properties even after processing them into valorized products (6). Before processing, potatoes are usually subjected to numerous pretreatments, among which blanching is one of the most important techniques (7). Blanching is an essential pretreatment for potato processing before frying, freezing, drying, and storing. The purpose of this is to prevent enzymatic browning by inactivating polyphenol oxidase, promoting a uniform color after frying, limiting oil absorption, and improving texture (8). Common techniques for blanching that have previously been applied to process vegetables include hot water blanching (WB), microwave blanching (MB), and steam blanching (SB). One of the oldest pretreatment techniques in potato processing is WB (65–85°C), which causes enzyme denaturation, reduces toxin production, and removes air from the tissue, as well as causes hydrolysis and solubilization of protopectin and gelatinization of starch granules (9). Challenges associated with water blanching include the consumption of large water volumes and the loss of nutrients. As exemplified in a study conducted by Ahmed et al. (10), the WB and SB of white cauliflower significantly reduced the phenolics (37.69% loss) and flavonoid content (43.42% loss) due to the breakdown of phenolics or losses (leached out) during cooking. Most of the bioactive compounds are relatively unstable to heat and get easily solubilized. Additionally, investigations revealed that SB required more energy (53.93 kJ mol^−1^) than hot-water treatment (41.41 kJ mol^−1^) to reach the transition state and then to unfold the polyphenol oxidase enzyme (11). To counter the problems associated with conventional blanching techniques, in recent years, there has been a significant increase in the use of microwave blanching in the field of food processing (10). It has several important benefits, including uniform treatment, improved penetrating power, and reduced nutritional losses. The concentration of phenols, the reduction in the number of microorganisms, and the antioxidant activity in vegetables have all been observed to be greatly increased by microwave blanching (12–14). Also, these different pretreatment methods could markedly reduce the amount of sugar, asparagine, and acrylamide in potato products (9). Though the effect of different blanching techniques has been explored earlier (2, 8, 15–18), the current study in a way is an extension of previous studies which aims to comparatively evaluate the effect of WB, SB, and MB on the structural and phytochemical compositions of colored potatoes in order to explore the most suitable method and to accomplish minimum losses. Efforts were made to investigate the effect of blanching techniques on the thermal and bioactive properties of potatoes. The current study will provide highly relevant information to food researchers and manufacturers so they can adopt a suitable blanching method as a pretreatment for different potato varieties.

## 2. Materials and methods

### 2.1. Materials and sample preparation

Potato varieties (purple potato variety) (Dry matter-18.30 ± 0.06%/Starch-13.20 ± 0.12%dry wt. basis) and *Lady Rosetta* (LR) (Dry matter-24.00 ± 0.12%/Starch-16.23 ± 0.16% dry wt. basis) were procured from the Department of Vegetable Science, Punjab Agricultural University, India. Potato tubers were sliced using stainless steel knife (thicknesses of slice6 ± 0.27 mm and diameter of 25 ± 0.52 mm). A vernier caliper was used to ascertain that the thickness of the slices remained within this range. After slicing they were blanched immediately to avoid enzymatic browning.

### 2.2. Blanching operations

Potato slices were subjected to WB (85°C for 5 min) as per the method adopted by Zhang et al. (18) and were then immediately cooled for 2 min by dipping them in cold water. Moisture on the surface was removed with the help of a paper towel. For SB, the slices were uniformly distributed in metal baskets and placed in an autoclave and steamed at 100°C for 5 min. MB was carried out as per the method used by Severini et al. (19), with some modifications. Potato slices were blanched in a microwave oven (Samsung, mc28h5025vr/tl, Malaysia) at the power level of 100, 300, and 900 W for 5 min and samples were coded as MB_100_, MB_300_, and MB_900_, respectively. Blanched samples were immediately dipped in water (20°C) to avoid over-cooking. Unblanched potato slices were taken as a control for both varieties, i.e., PP-1901 and LR.

### 2.3. Chemicals

2,2-diphenyl-1-picrylhydrazyl (DPPH), 2,2′- azino-bis (3-ethylbenzthiazoline-6-sulphonic acid) (ABTS), 6-hydroxy-2,5,7,8-tetramethyl chroman-2-carboxylic acid (Trolox), Folin–Ciocalteu reagent, gallic acid, sodium carbonate, aluminum chloride, potassium acetate, quercetin, acetic acid, hexane, metaphosphoric acid, and 2,6-dichlorophenol indophenol sodium salt (all chemicals of AR grade) were procured from Sisco Research Laboratories Pvt. Ltd. (SRL), Mumbai, India. Throughout the experiment, deionized water that had been filtered through a 0.22 μm filter was used.

### 2.4. Physicochemical analysis

#### 2.4.1. Moisture content and color analysis

For estimating moisture content, the standard hot air oven (Navyug Udyog, NU-101, India) method was used. Samples were placed inside the oven at 105°C for 24 h (20). Equation 1 was used to compute the moisture content (%):


(1) $$\text{Moisture content}\;\left(\%\right)=\frac{\text{Initial weight}-\text{Final weight}\;}{\text{weight of sample}}\times 100$$

Color parameters of potato samples were assessed using a handheld hunter lab colorimeter (MINOLTA, CM-508d, Japan) as per the method described by Aggarwal et al. (21). The samples were scanned in three distinct locations, and the results were presented as average of three repeat measurements. The browning index (22) of samples was calculated using the below-given equations.


(2) $$BI=\frac{100\left(x-0.31\right)}{0.17}$$

(3) $$\text{Where}\;x=\frac{a^{*}+1.75L*}{5.645L^{*}+a^{*}-3.012b*}$$

L*, a*, and b* represents lightness-darkness, redness-greenness, and yellowness-blueness.

#### 2.4.2. Qualitative and quantitative analysis of peroxidase test

Qualitative and quantitative peroxidase (POD) tests were performed for all the samples, as per the method adopted by Liburdi et al. (23), with slight modifications. For qualitative analysis, 1 g of potato sample in 3 ml of deionized water was crushed using a motor pestle, followed by filtration using Whatman filter paper No.4. Sample extract (1 ml) was then mixed with 1 ml of H_2_O_2_ (0.3%) and 0.5 ml of guaiacol solution (1%in absolute ethanol v/v). The solution was observed for any color development. The appearance of red color confirmed the presence of POD enzyme activity. For quantitative estimation, 5 g of potato slices were crushed in phosphate buffer (pH 7.8) containing 1% (w/v) polyvinyl/pyrrolidone. The crushed sample was cold centrifuged (Remi R-8C, India) at 4°C for 3,000 × g for 10 min. The supernatant (crude enzyme extract) obtained was used for the POD assay. In a final volume of 3.0 ml, the test mixture contained 50 mmol L^−1^ sodium phosphate buffer (pH 7.0), 12 mmol L^−1^ H_2_O_2_, 7 mmol L^−1^ guaiacol, and 0.1 ml of enzyme extract. UV Visible spectrophotometer (LABINDIA UV 3000, India) was used to record the increase in absorbance at 470 nm for 3 min. A change of absorbance from the initial reading to the final reading was taken as residual POD activity (Eq. 4).


(4) $$\text{Residual}\;\text{POD}\;\text{activity}\;\left(\%\right)=\frac{\text{Initial absorbance}}{\text{Final absorbance}}\times 100$$

### 2.5. Phytochemical analysis

#### 2.5.1. Extraction of bioactive compounds

The extraction method was adopted from the study by Wu et al. (24), with some modifications. In this method, 1 g of the sample was refluxed twice for 2 h with 80% methanol (w/v). All the samples were cooled to room temperature (24°C) before being filtered using Whatman filter paper No. 4 after extraction. The filtrate was diluted to a final volume of 100 ml with 80% methanol.

#### 2.5.2. Total phenolic and total flavonoid content

Total phenolic content (TPC) and total flavonoid content (TFC) were estimated as per the methods given by Fattahi et al. (25), with slight modifications. Using the Folin ciocalteau (FC) reagent, TPC in the samples was evaluated spectrophotometrically using a UV Visible spectrophotometer at 750 nm, and results were calculated using the Gallic acid equivalents (mg GAE/100 g of dry weight). Whereas TFC was determined by the aluminum chloride method and was calculated by comparing it to a quercetin calibration curve, and the results were expressed in mg of quercetin equivalent (mg QE/100 g).

#### 2.5.3. Ascorbic acid and total anthocyanins content

The titrimetric method was used to measure ascorbic acid content using 2,6-dichlorophenol indophenol dye (0.04% w/v) and results were expressed in mg/100 g (20). Anthocyanins were estimated in potato samples using the pH differential method as described by Albishi et al. (26). A measurement of 0.5 ml of extract was diluted with 2.5 ml of 0.4 M sodium acetate buffer (pH 4.5) and 2.5 ml of 0.025 M potassium chloride buffer (pH 1.0), separately. Taking distilled water as blank, the absorbance of diluted samples was noted after 15 min at 520 and 700 nm, respectively. Anthocyanin content (mg/100 g of dry matter) was measured using the below-given equation.


(5) $$\text{Anthocyanin content}=\frac{A\times DF\times MW}{W\times \varepsilon }$$

Where A is absorbance (A_520nm_–A_700nm_) (pH 1.0)–(A_520nm_–A_700nm_) (pH 4.5), DF is dilution factor, MW is the molecular weight of cyanidin-3-glucoside (449.2), W is the weight of the sample (g), and Ɛ is molar absorptivity (26,900).

#### 2.5.4. Total antioxidant activity

For the determination of antioxidant activity, DPPH radical scavenging activity (mg TE/100 g DW), ABTS radical scavenging activity (μg TE/g DW), and Ferric reducing antioxidant powers (FRAP) (mg FeSO_4_/100 g DW) were estimated as per the methods explained by Huang et al. (27), and the absorbance values were measured at 517, 734, and 593 nm, respectively. For reducing power assay (RPA) (mg AAE/100 g), metal chelating activity (MCA) (μmol EDTA/100 g) and hydroxyl radical scavenging activity (HRSA) (%inhibition) methods described by Asirvatham and Akhil (28) were used and absorbance was measured at 700, 562, and 532 nm. % Inhibition in the case of HRSA was calculated using below given equation.


(6) $$\%\text{Inhibition}=\frac{A_{C}-A_{S}}{A_{C}}\times 100$$

Where A_s_ and A_c_ depicts the absorbance of the sample and control, respectively.

### 2.6. Structural characterization

#### 2.6.1. Fourier transform infrared spectroscopy

Infrared spectra of blanched samples were recorded on an FTIR spectrophotometer (Agilent Cary 630 FTIR, Canada) (29). Directly following the background scan, blanched samples were placed on the diamond-attenuated total reflectance crystal. The sample was taken in such a quantity (10 mg) that it fully covered the crystal. After each sample analysis, the ATR crystal was carefully cleaned with isopropyl alcohol and dried with a soft tissue to remove any leftovers from the last sample. A total of 24 scans were conducted on each sample for the analysis, which was done in the 4,000–400 cm^−1^ range.

#### 2.6.2. Scanning electron microscopy analysis

A scanning electron microscope (JSM 6610-LV, JEOL, Japan, working at 5 kV) was used to morphologically analyze the control and MB_300_ at magnifications ranging from ×100 to ×5,000. The sample was mounted on an aluminum stub using double-backed cellophane tape after being coated with gold–palladium (60:40 w/w) in an auto fine coater, JEOL JFC-1600.

### 2.7. Texture profile analysis

A texture analyzer (LLOYD texture instrument LR 5 K, England) outfitted with a 50 kg load cell was used to determine the texture of fresh and treated potato cuboids. Samples were compressed to 50% (3 mm compression) of their original height (6 mm thickness), with a 5 s interval between compressions. The properties of hardness, springiness, cohesiveness, fracturability, and chewiness were measured for samples (21).

### 2.8. Thermal analysis

The differences in the thermal characteristics of the potato samples after each blanching treatment were examined using a differential scanning calorimeter (DSC) as mentioned in a study conducted by Zhang et al. (30), with slight modifications. A sealed crucible containing 10 mg of the material was placed within a DSC (DSC 200, NETZSCH, Germany), with a nitrogen purge flow of 20 mL/min. The crucible was heated inside the chamber at a rate of 10°C/min, with a temperature range of 25–120°C. To assess the potato starch’s thermal characteristics, the gelatinization enthalpy (H, expressed in J/g of dry matter), peak temperature (T_p_), onset temperature (T_o_), and conclusion temperature (T_c_) were calculated using a thermogram.

### 2.9. Statistical analysis

Experiments were replicated thrice for all the samples and the values were reported as mean ± standard deviation. Obtained results were statistically analyzed using Minitab 18.1 software and were subjected to two-way ANOVA (*p* < 0.05% significance level). To determine mean differences, the Tukey *post hoc* test was used. Points of FTIR were plotted using Origin Pro 2021 (version 9.8.0.200, OriginLab Corporation, USA).

## 3. Results and discussion

### 3.1. Impact of blanching on moisture content and color

Food quality, preservation, and resistance to degradation are all impacted by moisture content (31). The moisture content of all the potato samples was found to be approximately 74–76% (wet basis), with no significant difference in values (Table 1), indicating that there is no effect of blanching on moisture. *PP-1901* is a purple-colored potato variety containing anthocyanins (32), which are a group of naturally occurring phenolic compounds imparting color (33). In comparison to the control sample, the lightness of the purple potato significantly increased after blanching which might have happened due to the breakdown of anthocyanins at higher temperatures. A major increase in lightness was seen in the case of MB_900_ (67.01) as compared to the control (36.01), denoting a higher amount of anthocyanin disruption which is also confirmed in further sections. These results are consistent with past studies, which showed that the entire color change tended to increase as microwave power levels increased as natural pigments are unstable and sensitive to several elements, including light, temperature, oxidation, pH, metal ions, etc. (34, 35). On the other hand, for the LR variety, only WB increased the value of lightness whereas SB and MB decreased the lightness value. This could be a result of the significant amount of sugar leaching out in the WB samples, which exposed whiter surfaces and increased the lightness value (36). Another possible explanation can be a higher amount of anthocyanin deterioration in WB which is confirmed in further sections. Browning index (BI) is one of the crucial characteristics of products, which signifies the enzymatic or non-enzymatic browning occurring in the products (22). For the purple variety, all the samples except MB_100_ had a significant effect on the BI of the samples. The presence of residual enzymatic activity in the MB_100_ sample (Table 2) might be a possible explanation. Whereas a significant decrease in BI values for all LR variety samples was observed. It can be concluded that blanching techniques affect potatoes differently, depending on the variety treated.

**Table 1:** Effect of different blanching conditions on the quality parameters of PP-1901 and LR variety potatoes.

PP-1901 variety						
Samples	Control	WB	SB	MB_100_	MB_300_	MB_900_
Physico-chemical parameters						
Moisture content (%)	74 ± 1.4^A^	75.54 ± 1.52^A^	75.94 ± 0.59^A^	75.84 ± 2.1^A^	75.91 ± 1.66^A^	74.75 ± 2.7^A^
L*(lightness)	36.01 ± 2.01^C^	54.59 ± 2.03^B^	55.22 ± 1.86^B^	54.14 ± 1.46^B^	55.28 ± 1.86^B^	67.01 ± 2.93^A^
BI (Browning index)	20.80 ± 0.15^A^	11.86 ± 1.22^B^	4.54 ± 0.74^C^	19.89 ± 1.86^A^	4.50 ± 0.62^C^	9.15 ± 1.37^B^
Bioactive parameters						
Total phenols (mg GAE/100 g)	562.8 ± 20.7^C^	607.2 ± 20.9^B^	641.1 ± 27.4^B^	655.4 ± 39.5^B^	766.67 ± 15.94^A^	655.3 ± 20.7^B^
Total flavonoids (mg QE/100 g)	314.55 ± 3.48^A^	209.58 ± 2.27^B^	265.44 ± 2.86^D^	275.94 ± 2.79^BC^	283.94 ± 2.87^B^	269.87 ± 5.27^CD^
Ascorbic acid (mg/100 g)	19.38 ± 0.14^A^	9.11 ± 0.03^F^	11.7 ± 0.14^E^	17.47 ± 0.18^B^	15.52 ± 0.42^C^	12.54 ± 0.29^D^
Total Anthocyanins (mg/100 g)	180.45 ± 4.01^AB^	159.54 ± 4.51^C^	166.86 ± 2.58 ^BC^	176.25 ± 8.18 ^AB^	180.68 ± 5.68^A^	167.96 ± 2.91 ^ABC^
DPPH (mg TE/g DW)	8.09 ± 0.66^D^	11.32 ± 0.79^C^	11.51 ± 0.45^C^	15.02 ± 0.51^B^	18.27 ± 0.38^A^	11.59 ± 0.79^C^
RPA (mg AAE/100 g)	48.72 ± 2.48^E^	53.37 ± 1.26^D^	57.53 ± 0.79^C^	69 ± 0.87^B^	73.43 ± 1.26^A^	57.84 ± 0.37^C^
MCA (μmol EDTA/100 g)	25.95 ± 0.34^D^	27.15 ± 0.72^CD^	28.32 ± 0.81^BC^	26.26 ± 0.88^A^	31.66 ± 0.97^CD^	29.49 ± 0.71^B^
FRAP (mg FeSO_4_/100 g DW)	15.6 ± 0.45^D^	16.13 ± 0.68^CD^	17.46 ± 0.15^BC^	18.5 ± 0.26^B^	20.06 ± 0.41^A^	17 ± 0.72^C^
HRSA (%inhibition)	19.53 ± 0.45^C^	21.24 ± 0.95^C^	24.26 ± 1.1^B^	27.26 ± 1^A^	27.72 ± 1.19^A^	24.54 ± 1.1^B^
ABTS (μg TE/g DW)	7.4 ± 0.2^D^	9.66 ± 0.15^C^	12.72 ± 0.4^B^	13.65 ± 0.5^B^	15.71 ± 0.44^A^	12.72 ± 0.29^B^
LR variety						
Physico-chemical parameters						
Moisture content (%)	79.13 ± 0.87^A^	81.7 ± 1.15^A^	82.51 ± 1.86^A^	79.4 ± 1.1^A^	80.2 ± 1^A^	79.7 ± 1.26^A^
L*(lightness)	66.18 ± 1.2^B^	69.78 ± 0.38^A^	63.84 ± 0.36^C^	57.63 ± 0.7^D^	65.11 ± 0.67^BC^	66.94 ± 1.1^AB^
BI (Browning index)	34.58 ± 1.39^A^	12.98 ± 0.66^D^	13.99 ± 0.5^D^	25.04 ± 0.83^B^	10.07 ± 0.27^E^	21.07 ± 0.83^C^
Bioactive parameters						
Total phenols (mg GAE/100 g)	166.37 ± 3.56^CD^	173 ± 3.58^BCD^	183.7 ± 4.09^B^	176.99 ± 3.56^BC^	214.51 ± 5.64^A^	165.44 ± 3.68^D^
Total flavonoids (mg QE/100 g)	26.12 ± 0.82^D^	28.5 ± 1.25^CD^	31.2 ± 0.86^BC^	33.58 ± 1.33^AB^	35.72 ± 0.47^A^	32.07 ± 0.98^B^
Ascorbic acid (mg/100 g)	14.65 ± 0.38^A^	10.24 ± 0.29^D^	9.53 ± 0.25^D^	13.59 ± 0.36^B^	12.56 ± 0.28^C^	8.45 ± 0.27^E^
Total Anthocyanins (mg/100 g)	23.48 ± 0.32^A^	18.72 ± 0.28^D^	19.52 ± 0.42^CD^	21.58 ± 0.76^B^	20.79 ± 0.63^BC^	19.95 ± 0.42^CD^
DPPH (mg TE/g DW)	2.18 ± 0.04^D^	2.39 ± 0.08^C^	2.45 ± 0.07^C^	3.24 ± 0.02^B^	3.46 ± 0.02^A^	2.51 ± 0.08^C^
RPA (mg AAE/100 g)	27.46 ± 0.4^F^	36.63 ± 0.49^E^	39.49 ± 0.05^D^	48.88 ± 1.07^B^	53.07 ± 0.61^A^	46.48 ± 0.45^C^
MCA (μmol EDTA/100 g)	6.23 ± 0.01^E^	7.56 ± 0.04^D^	8.62 ± 0.25^C^	10.08 ± 0.02^B^	12.6 ± 0.15^A^	9.92 ± 0.04^B^
FRAP (mg FeSO4/100 g)	4.92 ± 0.05^D^	5.91 ± 0.1^C^	6.28 ± 0.15^C^	7.66 ± 0.28^B^	8.6 ± 0.4^A^	7.58 ± 0.32^B^
HRSA (%inhibition)	8.45 ± 0.2^E^	8.77 ± 0.1^DE^	9.08 ± 0.03^D^	9.79 ± 0.16^C^	11.67 ± 0.27^A^	10.5 ± 0.17^B^
ABTS (μg TE/g DW)	2.58 ± 0.02^F^	3.07 ± 0.05^E^	3.77 ± 0.09^D^	4.49 ± 0.02^B^	5.16 ± 0.06^A^	4.08 ± 0.05^C^

Mean ± SD (*n* = 3) that do not share a superscript letter in a column are significantly different (*p* < 0.05).

**Table 2:** Effect of different blanching conditions on residual POD activity of PP-1901 and LR variety potatoes.

Blanching	Residual POD activity (PP-1901)		Residual POD activity (LR)	
	Qualitative	Quantitative (Residual activity)	Qualitative	Quantitative (Residual activity)
Control	+	100 ± 0.00^A^	+	100 ± 0.00^A^
WB	−	8.59 ± 0.31^C^	−	8.90 ± 0.06^C^
SB	−	7.49 ± 0.06^D^	−	7.57 ± 0.17^D^
MB_100_	+	32.28 ± 0.59^B^	+	43.11 ± 0.55^B^
MB_300_	−	6.53 ± 0.32^E^	−	7.36 ± 0.38 ^DE^
MB_900_	−	5.85 ± 0.10^E^	−	6.68 ± 0.06^E^

Mean ± SD (*n* = 3) that do not share a superscript letter in a column are significantly different (*p* < 0.05); POD- Peroxidase enzyme activity.

### 3.2. Impact of blanching on residual peroxidase activity

The oxidation of phenolic compounds by oxidoreductase enzymes results in the enzymatic browning reaction in potatoes which is undesirable during potato processing (37). POD is the most heat-resistant oxidoreductase enzyme found in vegetables which are utilized as marker enzymes to gauge the effectiveness of blanching. Other less heat-resistant enzymes are also killed if there is no trace of POD activity (38). A significant reduction (*p* < 0.05) in residual POD activity was observed in all blanched samples with MB_900_ showing maximum reduction, indicating the intensity of enzymatic denaturation brought by high-power microwaves (Table 2). At higher power, microwaves generate thermal stresses which lead to modification of the secondary structure of enzymes, thus leading to a decline in their activity (39). MB_100_ samples for both varieties showed a positive residual POD activity (qualitative analysis) which can be attributed to the lower amount of thermal shock experienced by POD at a lower microwave power. These findings are in agreement with those observed by Wang et al. (40) where with an increase in microwave power, the amount of POD in red bell pepper decreased. A similar kind of observation was made by Liu et al. (41) where microwave blanching rapidly and effectively inactivated POD in sweet potatoes. This was attributed to the heating of the core and surface simultaneously thereby causing higher degradation. Differences in residual POD activity for different blanching techniques can be attributed to the difference in the mode of action.

### 3.3. Impact of blanching on phytochemicals

Polyphenols possess antioxidant properties that enable them to scavenge the formation of free radicals and prevent several oxidative reactions by self-oxidation (42). Blanching being one of the most important hydrothermal processes used in potato processing is useful for the inactivation of enzymes which prevents the oxidation of desired polyphenols present in potatoes thus reducing nutrient loss and improving food quality (9).

Fresh potato tubers had a TPC value of 562.8 ± 20.7 and 166.37 ± 3.56 mg GAE/100 g dry basis (d.b.) for purple and LR variety, respectively (Table 1), which were similar to the values reported in the previous studies performed by Burgos et al. (43) and D’amelia et al. (44). WB potatoes of the purple variety showed 7.8% increase in TPC while 10.41% increase was noticed in the LR variety. Similarly, a previous study on water blanching of African nightshade (African leafy vegetable) also showed an increase in phenolic content (45) and the authors reported that heat-mediated disarrangement of the polyphenol-protein complexes had favored the extraction of the polyphenols. Tubers subjected to SB and MB also showed a significant increase in TPC. For SB, the TPC increased to 13.9% for purple and 6.38% for LR variety (Table 1). Heras-Ramírez et al. (46) also observed an increase in phenolic content for blanched apples which was attributed to enzyme inactivation. With an increase in microwave power from 100 to 300 W, MB slices showed an increasing TPC over time, but the increase was only sustained up to 300 W. Subsequent increases in microwave power (900 W) resulted in a TPC reduction of 14.9% for the purple variety and 22.88% for the LR variety. The increase in microwave power elevates electrical energy absorbed into the solution and the material which causes the mobility of polar molecules to increase, which raises the temperature of the solution and speeds up the breakdown process (47). Furthermore, variation in TPC values as per microwave power might be the result of an increase in phenolic content induced by cell injury, whereas a decrease in TPC could be due to heat generation at 900 W and residual POD activity at 100 W.

TFC in fresh potatoes was found to be 314.55 ± 3.48 and 26.12 ± 0.82 mg QE/100 g d.b. for the purple and LR varieties, respectively. For the LR variety, the TFC value increased for all treated samples in comparison to the control, but the value decreased from MB_300_ W to MB_900_ W, which can have a similar explanation to that of TPC. In the case of the purple variety, a decrease in TFC was observed. The difference in the results among varieties may be due to the more heat-labile flavonoids (anthocyanins) present in the purple variety (48). Depending on how they are structurally constructed, flavonoids can be sensitive to heat treatment. The biological activity of flavonoids and their stability are both influenced by temperature (49).

Blanching significantly (*p* < 0.05) lowered the ascorbic acid content (AAC) for treated potato samples. Out of all samples, WB potatoes had the least retention of AAC in comparison to control samples and had a reduced rate of 52.99 and 36.92% for purple and LR varieties, respectively. Leaching of soluble particles into the blanching water, which has been viewed as the primary drawback of WB, could be one possible explanation for this loss (50). Significant ascorbic acid losses through water blanching of potatoes have also been reported by Ambe Desmond et al. (51). Similar to conventional blanching methods utilized in the study, microwave blanching also resulted in a decrease in AAC, but the decrease was less pronounced. The outcomes are consistent with earlier research by Xanthakis et al. (52), which demonstrated that microwave blanching considerably retained ascorbic acid concentration in mangoes as compared to traditional blanching. Upon increasing microwave power from 100 to 900 W, there was a reduction in AAC of the blanched potato slices due to the electromagnetic waves assisted destruction of vitamin C. Likewise, E. Abano (53) observed that microwave drying of sweet potato slices resulted in a decrease in ascorbic acid content. Due to its high heat sensitivity and reactivity, ascorbic acid primarily degrades through thermal or oxidative processes (54).

Blanching techniques significantly decreased the total anthocyanin content in both varieties. However, MB_300_ was found to be the most suitable blanching method to retain anthocyanins in both varieties. These results are in accordance with the previous study of McDougall et al. (55), in which the anthocyanin losses increased with blanching time, and the amount of anthocyanins retained was dependent on the blanching regime. According to the author, prolonged temperature treatments resulted in greater anthocyanin losses while blanching times of 2 to 5 min helped to preserve anthocyanins in rhubarb.

One of the most significant and well-researched properties associated with phenolics, flavonoids, ascorbic acid, and anthocyanins is their antioxidant ability (56). Different techniques, including the single electron transfer method (DPPH, RPA, FRAP, and ABTS) and hydrogen atom transfer methods (MCA and HRSA) were used to assess the antioxidant capacity of potato samples (Table 1). With the hydrogen atom transfer technique, the antioxidant activity was quite high which could be attributed to the structural characteristics of the phenolic constituents in colored potatoes and the different mechanisms of the antioxidant assays. Additionally, anthocyanin uses the hydrogen atom transfer pathway to exert its antioxidant properties (57).

It is evident from the data that all three blanching techniques can increase the antioxidant activity of potatoes when compared to untreated samples; the lowest increase was found in WB samples for both varieties. A similar conclusion has been reported by Marzuki et al. (58), and the antioxidant such as ascorbic acid and phenolic compounds released into the hot water is probably responsible for the less increase in the antioxidant capacity of WB samples. Additionally, MB_300_-treated samples had greater total antioxidant activity values than all other samples which could be because microwaves can break down the vegetable cell walls, making it easier for antioxidants to release (e.g., phenolic compounds) while this phenomenon is not that effective at lower power levels, whereas higher powers are destructive due to high heat generation (59). From the above results, it can be concluded that MB_300_ is the most suitable blanching method for retaining the phytochemicals present in both potato varieties.

### 3.4. Impact of blanching on functional groups

**Figure 1:**
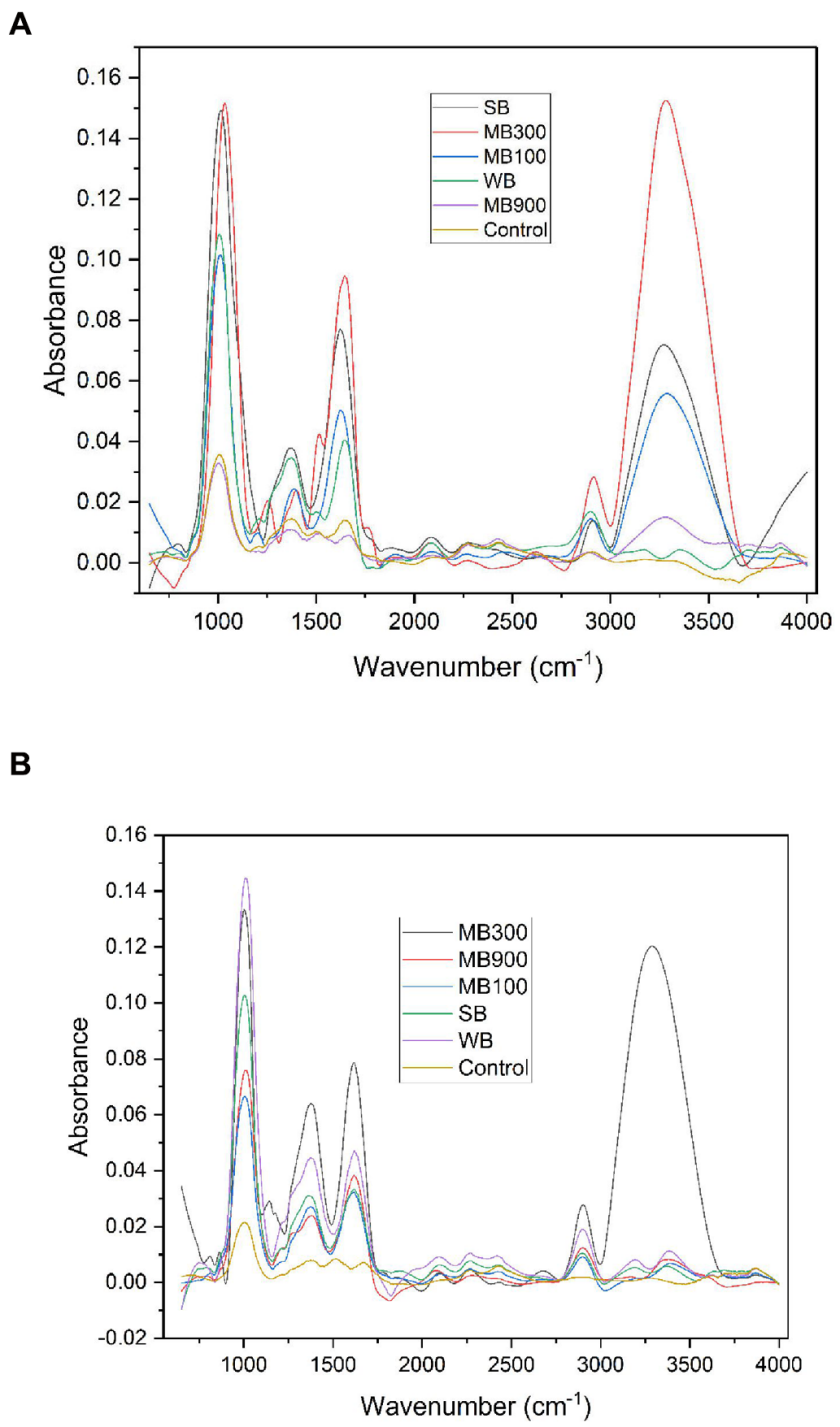
FTIR spectrum of **(A)** LR varety and **(B)** PP-1901 variety (WB-Hot water blanching, SB-Steam blanching, MB_100_^−^ Microwave blanching at 100 W, MB_300_^−^ microwave blanching at 300 W and MB_900_^−^ Microwave blanching at 900w).

To determine the level of alteration and interaction of functional groups following different blanching treatments, IR spectral analysis of control and blanched potato samples was performed. Any change in the functional groups was interpreted as a result of tissue disintegration produced by the heat action of blanching. FTIR spectra of PP-1901 and LR variety are shown in Figure 1. Vibrational peaks ranging between 1,600 and 1700 cm^−1^ (C=O stretching vibrations) indicate the presence of amide I, 1480–1,575 cm^−1^ (N-H in-plane bending vibration) amide II, and 1,200–1,400 cm^−1^ (N-H deformation and C-N stretching) amide III region. Bands with wavenumber 3,200–3,400 cm^−1^ and 2,800–3,000 cm^−1^ represent -OH and -CH stretching vibrations, respectively. Absorption around 2,921 cm^−1^ depicts CH_3_ asymmetric stretching vibrations in aliphatic chains of protein (60). These results support an increase in the bioactive parameter value discussed previously. The absorption bands around 3,300–3,600, 2,900, 1,150, and 1,000–1,100 cm^−1^ are due to starch present which has an OH, C-H, C-O-C, and C-O functional group. Furthermore, the distinctive C-O-C ring vibration on starch results in an absorbance peak of approximately 700–900 cm^−1^. The OH group’s C-O bending resulted in an absorbance peak at roughly 1,648 cm^−1^ (61). An increase in the intensity of peaks after blanching indicates an increase in the amount of functional group linked with the molecular bond (for LR variety at 3854 cm^−1^, the intensity increased from 0.02083 of control to 0.12553 of MB_300_ sample and for PP 1901 at 989 cm^−1^, the intensity increased from 0.04302 of control to 0.41350 of MB_300_ sample). The O-H bonds (also known as alcohol bonds), which are frequently present in both native and hydrolyzed potato starch, can be seen in peaks between 3,100 and 3,700 cm^−1^. The starch molecules’ CH_2_ bonds (methylene linkages), which are missing from native potato starch but present in hydrolyzed potato starch, are represented by the wavenumber between 2,920 and 2,928 cm^−1^ (62).

### 3.5. Impact of blanching on microstructure

**Figure 2:**
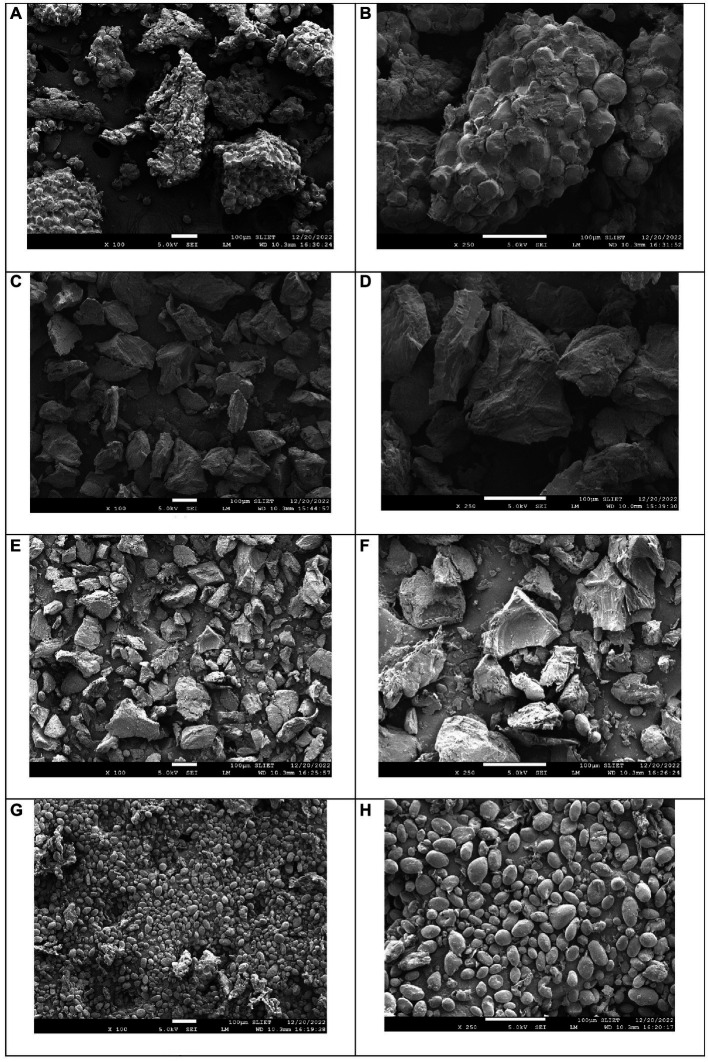
SEM images of control LR at **(A)** × 100 and **(B)** × 250 magnification; MB_300_ LR variety **(C)** × 100 and **(D)** × 250 magnification; control PP-1901 at **(E)** × 100 and **(F)** × 250 magnification; and MB_300_ at **(G)** × 100 and **(H)** × 250 magnification.

The morphology of the potato parenchyma cell before and after MB_300_ blanching under a Scanning electron microscope is shown in Figure 2. It can be observed that control samples of both samples exhibited birefringence which was observed at different magnifications. MB_300_ significantly affected the microstructure of both the LR and purple potatoes, as shown in Figures 2D,H. Also, there was a significant difference between the structural conformity of both varieties. This is due to the presence of a high amount of dry matter and total starch content in the LR variety than in the purple variety due to which the swelling and cell wall distension were more evident after blanching in the former one (63). Cell sloughing or swelling mainly occurs in potatoes with high dry matter content. A similar kind of disruption in potato microstructure after radiofrequency blanching was observed by Zhang et al. (9), which was attributed to blanching-induced starch gelatinization and cell disruption. Similarly, in a study conducted by Gomide et al. (64), microwave drying significantly affected the microstructure of potato samples. A possible explanation for this was that microwaves caused volumetric heating in the sample and led to a reduction in firmness and gelatinized starch. The decrease in values of textural parameters of both LR and purple potato varieties discussed in the next sections further supports these results. Additionally, because of the rapid evaporation during microwave blanching, a porous matrix with a more compact structure was created. However, the purple-colored swollen starch granules diffused through the cell wall, causing it to lose its original polyhedral shape; in contrast, in LR, the area of the wall expanded as a result of the cell wall distension and swelling of the gelatinized starch. Potato microstructure was significantly altered by microwave blanching although the impact is dependent on the variable granule architecture (crystalline to amorphous ratio) and the various properties of starch, which might differ dramatically among potato varieties.

### 3.6. Impact of blanching on textural properties

Texture is one of the most essential quality features of foods. Foods have varying textural qualities which are caused by inherent differences due to differences in variety, differences in the developmental stage, and differences caused by processing procedures (65). Blanching had a significant effect (*p* < 0.05) on the textural properties of control and blanched samples, as shown in Table 3. Values of hardness and springiness decreased for both LR and PP-1901 varieties after blanching for all the different treatments used. MB_900_ samples had the lowest value of textural parameters, indicating larger tissue damage to potatoes. A similar kind of reduction in values of hardness, chewiness, and fracturability of radio frequency blanched potato cuboids was observed by Zhang et al. (9). This was attributed to water loss that led to softening of the texture after blanching. Likewise, Jiang et al. (66) observed a decrease in these values for water and radio-frequency blanched sweet potato. This occurrence was attributed to cell wall integrity damage, which might have impacted texture qualities.

**Table 3:** Effect of different blanching conditions on the textural properties of PP-1901 and LR variety potatoes.

Variety	Treatment	Treatment	Hardness (g)	Fracturability (g)	Springiness	Cohesiveness	Chewiness
PP-1901	Control	Unblanched	751.09 ± 13.34^A^	720.19 ± 2.20^A^	0.91 ± 0.03^A^	0.12 ± 0.02^A^	96.89 ± 2.33^C^
	MB	100 W	387.67 ± 10.44^B^	442.47 ± 17.02^B^	0.08 ± 0.01^C^	0.077 ± 0.003^B^	342.51 ± 16.11^B^
		300 W	74.27 ± 4.24^E^	0^C^	0.2 ± 0.1^B^	0.061 ± 0.002^B^	46.077 ± 1.41^D^
		900 W	15.56 ± 0.88^F^	0^C^	0.06 ± 0.004^C^	0.048 ± 0.002^B^	5.11 ± 0.07^E^
	SB	100(°C) for 5 min	118.63 ± 2.88^D^	0^C^	0.11 ± 0.005^BC^	0.08 ± 0.005^B^	111.72 ± 0.16^C^
	WB	85(°C) for 5 min	350.55 ± 6.29^C^	0^C^	0.120.006^BC^	0.06 ± 0.03^B^	432.21 ± 7.42^A^
LR	Control	Unblanched	714.62 ± 5.31^A^	728.59 ± 2.31^A^	0.92 ± 0.02^A^	0.25 ± 0.02^A^	101.84 ± 1.58^E^
	MB	100 W	426.42 ± 11.43^B^	485.28 ± 16.03^B^	0.12 ± 0.04^BC^	0.12 ± 0.02^BC^	491.3 ± 32.3^B^
		300 W	252.34 ± 5.9^C^	0^C^	0.08 ± 0.007^C^	0.1 ± 0.07^BC^	164.12 ± 10.56^D^
		900 W	105.99 ± 7.95^D^	0^C^	0.08 ± 0.005^C^	0.06 ± 0.02^C^	42.01 ± 5.7^F^
	SB	100(°C) for 5 min	247.7 ± 16.15^C^	0^C^	0.07 ± 0.02^C^	0.15 ± 0.03^ABC^	306.1 ± 17.6^C^
	WB	85(°C) for 5 min	418.62 ± 11.45^B^	0^C^	0.16 ± 0.015^B^	0.19 ± 0.015^AB^	1196.2 ± 36^A^

Mean ± SD (*n* = 3) that do not share a superscript letter in a column are significantly different (*p* < 0.05).

### 3.7. Impact of blanching on thermal properties

For analyzing melting, crosslinking, and detection of water loss from the food matrix, crystallization, and gelatinization, thermal analysis utilizing DSC is frequently used (29). Starch is principally responsible for the thermal characteristics of potatoes. However, the thermal properties of potatoes are also influenced by their non-starch polysaccharide and protein (30). The gelatinization temperature (T_g_) and enthalpy (∆H) of control and blanched samples of both varieties are shown in Table 4. T_g_ and ∆H of control for LR and PP-1901 samples were found to be 60 and 63°C and 1.69 and 2.73 J/g, respectively. A similar T_g_ value (60°C) for raw potatoes has been reported by Tian et al. (67). It was observed that blanching significantly affected (*p* < 0.05) both T_g_ and ∆H. Disruption in the ordered structure of potato tissue which has been confirmed in previous sections might have resulted in decreasing both T_g_ and ∆H values of potato samples. A decrease in the value of T_g_ indicates a loss in molecular order and crystalline structure (68). A decrease in ∆H values after blanching indicates an increase in the degree of gelatinization. ∆H provides an overall measure of crystallinity as a measure of the loss of molecular order inside the granule; the larger value of enthalpy denotes a lower proportion of gelatinized starch (67). Potato starch has B-type crystallinity which when subjected to hydrothermal treatments disrupts its crystalline structure and thereby increases its digestibility (62). The difference in varieties, growing conditions, and physicochemical composition can also affect the gelatinization properties of potatoes (69). Similarly, processing techniques like boiling and cooling, as discussed by Tian et al. (67), irradiation (70), and radio frequency blanching (30) affected the thermal properties of potatoes.

**Table 4:** Effect of different blanching conditions on thermal properties of PP-1901 and LR variety potatoes.

Variety	Sample	T_g_ (°C)	∆H (J/g)
LR	Control	60.11 ± 1.94^A^	1.69 ± 0.02^A^
	MB_100_	48.39 ± 0.62^B^	1.75 ± 0.03^A^
	MB_300_	59.63 ± 0.5^A^	1.58 ± 0.17^A^
	MB_900_	47.73 ± 0.89^B^	1.34 ± 0.05^B^
	WB	43.79 ± 1.19^C^	1.18 ± 0.09^B^
	SB	44.37 ± 0.99^C^	1.67 ± 0.03^A^
PP-1901	Control	63.36 ± 1.92^A^	2.73 ± 0.04^A^
	MB_100_	51.79 ± 1.24^B^	1.47 ± 0.04^D^
	MB_300_	62.58 ± 0.6^A^	2.31 ± 0.08^B^
	MB_900_	44.08 ± 0.8^C^	1.73 ± 0.05^C^
	WB	46.14 ± 0.8^C^	1.6 ± 0.06^CD^
	SB	52.21 ± 1.32^B^	2.24 ± 0.07^B^

Mean ± SD (*n* = 3) that do not share a superscript letter in a column are significantly different (*p* < 0.05).

## 4. Conclusion

The results showed that blanching significantly affected the bioactive properties and antioxidant potential of colored potatoes. In comparison to conventional blanching methods, microwave blanching was the most suitable technique to retain flavonoids, anthocyanins, phenolics, and ascorbic acid. More than 15% of phytochemicals increased at different power levels. Our research found that 300 W power was the most suitable blanching process parameter, which was also supported by the FTIR results. The difficulty encountered at a high-power level (900 W) was related to increased heat generation, which impacted heat-labile phytochemicals found in potatoes. On the other hand, a lower power level (100 W) was ineffective to remove residual POD levels. As a result, the databases employed in the current study provided a broad overview of the blanching methods. An appropriate approach should have a high capacity for inactivating the browning enzyme while retaining the material’s original color and nutritional value. These process parameters could also be used to facilitate the production of other related products like French fries and other processed commodities, which has a huge commercial potential.

## Data availability statement

The original contributions presented in the study are included in the article/supplementary material, further inquiries can be directed to the corresponding authors.

## Author contributions

RS: conceptualization, data curation, writing–original draft, preparation, methodology, visualization, and writing–review and editing. SK: supervision, conceptualization, writing–original draft and preparation, methodology, and writing–review and editing. PA: editing and revising. AD: supervision, conceptualization, software, writing–original draft and preparation, methodology, and writing–review and editing.

## Conflict of interest

The authors declare that the research was conducted in the absence of any commercial or financial relationships that could be construed as a potential conflict of interest.

## Publisher’s note

All claims expressed in this article are solely those of the authors and do not necessarily represent those of their affiliated organizations, or those of the publisher, the editors and the reviewers. Any product that may be evaluated in this article, or claim that may be made by its manufacturer, is not guaranteed or endorsed by the publisher.

## References

[1] Pedreschi F. Fried and dehydrated potato products., advances in potato chemistry and technology. Elsevier. (2009) 319–37. Available at: https://www.sciencedirect.com/science/article/abs/pii/B9780123743497000118

[2] Okonkwo CE Moses OI Nwonuma C Abiola T Benjamin BO Folorunsho JO. Infrared and microwave as a dry blanching tool for Irish potato: product quality, cell integrity, and artificial neural networks (ANNs) modeling of enzyme inactivation kinetic. Innov Food Sci Emerg Technol. (2022) 78:103010. doi: 10.1016/j.ifset.2022.103010

[3] Sharma S Jaiswal AK Jaiswal S. Potato In: Jaiswal AK, editor. Nutritional Composition and Antioxidant Properties of Fruits and Vegetables. Amsterdam: Elsevier (2020, 347). 339.

[4] Camire ME Kubow S Donnelly DJ. Potatoes and human health. Crit Rev Food Sci Nutr. (2009) 49:823–40. doi: 10.1080/10408390903041996, PMID: 19960391

[5] Lachman J Hamouz K. Red and purple coloured potatoes as a significant antioxidant source in human nutrition - a review. Plant Soil Environ. (2005) 51:477–82. doi: 10.17221/3620-PSE

[6] Nayak B Berrios J De J Powers JR Tang J Ji Y. Colored potatoes (*Solanum tuberosum* L.) dried for antioxidant-rich value-added foods. J Food Process Preserv. (2011) 35:571–80. doi: 10.1111/j.1745-4549.2010.00502.x

[7] Tekalign T. Processing quality of improved potato (*Solanum tuberosum* L.) cultivars as influenced by growing environment and blanching. African J Food Sci. (2011) 5:324–32. Available at: https://academicjournals.org/journal/AJFS/article-full-text-pdf/71031A23523.pdf

[8] Amaral RDA Achaerandio I Benedetti BC Pujolà M. The influence of edible coatings, blanching and ultrasound treatments on quality attributes and shelf-life of vacuum packaged potato strips. LWT Food Sci Technol. (2017) 85:449–55. doi: 10.1016/j.lwt.2017.03.062

[9] Zhang Z Guo C Gao T Fu H Chen Q Wang Y. Pilot-scale radiofrequency blanching of potato cuboids: heating uniformity. J Sci Food Agric. (2018) 98:312–20. doi: 10.1002/jsfa.8473, PMID: 28585268

[10] Ahmed FA Ali RFM. Bioactive compounds and antioxidant activity of fresh and processed white cauliflower. Biomed Res Int. (2013) 2013:1–9. doi: 10.1155/2013/367819, PMID: 24171164 PMC3793502

[11] Moscetti R Raponi F Monarca D Bedini G Ferri S Massantini R. Effects of hot-water and steam blanching of sliced potato on polyphenol oxidase activity. Int J Food Sci Technol. (2019) 54:403–11. doi: 10.1111/ijfs.13951

[12] Nie J Chen D Lu Y Dai Z. Effects of various blanching methods on fucoxanthin degradation kinetics, antioxidant activity, pigment composition, and sensory quality of Sargassum fusiforme. Lwt. (2021) 143:111179. doi: 10.1016/j.lwt.2021.111179

[13] Li B Kimatu BM Pei F Chen S Feng X Hu Q. Non-volatile flavour components in Lentinus edodes after hot water blanching and microwave blanching. Int J Food Prop. (2018) 20:S2532–42. doi: 10.1080/10942912.2017.1373667

[14] Khan MK Ahmad K Hassan S Imran M Ahmad N Xu C. Effect of novel technologies on polyphenols during food processing. Innov Food Sci Emerg Technol. (2018) 45:361–81. doi: 10.1016/j.ifset.2017.12.006, PMID: 37042630

[15] Budi S Abduh M Leong SY Zhao C Baldwin S Burritt DJ. Kinetics of Colour Development During Frying of Potato Pre-Treated with Pulsed Electric Fields and Blanching: Effect of Cultivar. *Foods* (2021) 10.3390/foods10102307 PMC8535209 34681356

[16] Al-khusaibi MK Niranjan K. The Impact of Blanching and High-Pressure Pretreatments on Oil Uptake of Fried Potato Slices. Food Bioprocess Technol. (2012) 5:2392–400. doi: 10.1007/s11947-011-0562-2

[17] Wu B Guo Y Wang J Pan Z Ma H. Effect of thickness on non-fried potato chips subjected to infrared radiation blanching and drying. J Food Eng. (2018) 237:249–55. doi: 10.1016/j.jfoodeng.2018.05.030

[18] Zhang Y Kahl DHW Bizimungu B Lu ZX. Effects of blanching treatments on acrylamide, asparagine, reducing sugars and colour in potato chips. J Food Sci Technol. (2018) 55:4028–41. doi: 10.1007/s13197-018-3329-1, PMID: 30228401 PMC6133836

[19] Severini C Baiano A De Pilli T Carbone BF Derossi A. Combined treatments of blanching and dehydration: study on potato cubes. J Food Eng. (2005) 68:289–96. doi: 10.1016/j.jfoodeng.2004.05.045

[20] Gaithersburg M. AOAC Official Methods of Analysis (Methods 967.21). Rockville, MD: AOAC International (2000).

[21] Aggarwal P Kaur S Kaur N. Intermediate moisture kinnow bar from low grade kinnow mandarins: Phytonutritional profile, morphological characterization, and storage stability. Food Biosci. (2022) 49:101837. doi: 10.1016/j.fbio.2022.101837

[22] Dibanda RF Akdowa EP Tongwa QM. Effect of microwave blanching on antioxidant activity, phenolic compounds and browning behaviour of some fruit peelings. Food Chem. (2020) 302:125308. doi: 10.1016/j.foodchem.2019.125308, PMID: 31401419

[23] Liburdi K Benucci I Esti M. Effect of microwave power and blanching time in relation to different geometric shapes of vegetables. LWT Food Sci Technol. (2019) 99:497–504. doi: 10.1016/j.lwt.2018.10.029

[24] Wu T Yan J Liu R Marcone MF Aisa HA Tsao R. Optimization of microwave-assisted extraction of phenolics from potato and its downstream waste using orthogonal array design. Food Chem. (2012) 133:1292–8. doi: 10.1016/j.foodchem.2011.08.002

[25] Fattahi S Zabihi E Abedian Z Pourbagher R Motevalizadeh Ardekani A Mostafazadeh A. Total phenolic and flavonoid contents of aqueous extract of stinging nettle and in vitro antiproliferative effect on Hela and BT-474 cell lines. Int J Mol Cell Med. (2014) 3:102–7. PMID: 25035860 PMC4082812

[26] Albishi T John JA Al-Khalifa AS Shahidi F. Phenolic content and antioxidant activities of selected potato varieties and their processing by-products. J Funct Foods. (2013) 5:590–600. doi: 10.1016/j.jff.2012.11.019

[27] Huang H Sun Y Lou S Ye X. *In vitro* digestion combined with cellular assay to determine the antioxidant activity in Chinese bayberry (*Myrica rubra* Sieb. Et Zucc.) fruits: a comparison with traditional methods. Food Chem. (2014) 146:363–70. doi: 10.1016/j.foodchem.2013.09.071, PMID: 24176355

[28] Asirvatham R Akhil J. Evaluation of *in vitro* and *in vivo* anti-oxidant potential of Morinda Reticulata gamble tubers in Wistar albino rats subjected to CCL 4 and paracetamol induced hepatotoxicity. Indones J Pharm. (2017) 28:147–57. doi: 10.14499/indonesianjpharm28iss3pp147

[29] Dhiman A Suhag R Verma K Thakur D Kumar A Upadhyay A. Influence of microfluidization on physico-chemical, rheological, thermal properties and cholesterol level of cow ghee. LWT. (2022) 160:113281. doi: 10.1016/j.lwt.2022.113281

[30] Zhang Z Yao Y Shi Q Zhao J Fu H Wang Y. Effects of radio-frequency-assisted blanching on the polyphenol oxidase, microstructure, physical characteristics, and starch content of potato. Lwt. (2020) 125:109357. doi: 10.1016/j.lwt.2020.109357

[31] Nielsen SS. Phenol-sulfuric acid method for total carbohydrates. Food Anal Lab Manual Springer. (2010) 47–53. doi: 10.1007/978-1-4419-1463-7_6

[32] Nayak B Liu RH Berrios JDJ Tang J Derito C. Bioactivity of antioxidants in extruded products prepared from purple potato and dry pea flours. J Agric Food Chem. (2011) 59:8233–43. doi: 10.1021/jf200732p, PMID: 21615124

[33] Qiu G Wang D Song X Deng Y Zhao Y. Degradation kinetics and antioxidant capacity of anthocyanins in air-impingement jet dried purple potato slices. Food Res Int. (2018) 105:121–8. doi: 10.1016/j.foodres.2017.10.050, PMID: 29433199

[34] Szadzińska J Łechtańska J Kowalski SJ Stasiak M. The effect of high power airborne ultrasound and microwaves on convective drying effectiveness and quality of green pepper. Ultrason Sonochem. (2017) 34:531–9. doi: 10.1016/j.ultsonch.2016.06.030, PMID: 27773279

[35] Su Y Zhang M Bhandari B Zhang W. Enhancement of water removing and the quality of fried purple-fleshed sweet potato in the vacuum frying by combined power ultrasound and microwave technology. Ultrason Sonochem. (2018) 44:368–79. doi: 10.1016/j.ultsonch.2018.02.049, PMID: 29680623

[36] Bingol G Wang B Zhang A Pan Z McHugh TH. Comparison of water and infrared blanching methods for processing performance and final product quality of French fries. J Food Eng. (2014) 121:135–42. doi: 10.1016/j.jfoodeng.2013.08.001

[37] Erihemu WM Zhang F Wang D Zhao M Cui N Gao G. Optimization of the process parameters of ultrasound on inhibition of polyphenol oxidase activity in whole potato tuber by response surface methodology. LWT. (2021) 144:111232. doi: 10.1016/j.lwt.2021.111232

[38] Fellows P. Principles and practice In: Fellows PJ, editor. Food Process Technology. 2nd ed. Chickester, UK: Ellis Horwood (2000). 369–80.

[39] Zhang C Lyu X Muhammad R Tong Y Zhao W Yang R. Microwave heating instead of blanching to produce low-fat French fries. Innov Food Sci Emerg Technol. (2023) 84:103298. doi: 10.1016/j.ifset.2023.103298

[40] Wang J Yang X-H Mujumdar AS Wang D Zhao J-H Fang X-M. Effects of various blanching methods on weight loss, enzymes inactivation, phytochemical contents, antioxidant capacity, ultrastructure and drying kinetics of red bell pepper (*Capsicum annuum* L.). LWT. (2017) 77:337–47. doi: 10.1016/j.lwt.2016.11.070

[41] Liu P Mujumdar AS Zhang M Jiang H. Comparison of three blanching treatments on the color and anthocyanin level of the microwave-assisted spouted bed drying of purple flesh sweet potato. Dry Technol. (2015) 33:66–71. doi: 10.1080/07373937.2014.936558

[42] Wei P Zhu K Cao J Dong Y Li M Shen X. The inhibition mechanism of the texture deterioration of tilapia fillets during partial freezing after treatment with polyphenols. Food Chem. (2021) 335:127647. doi: 10.1016/j.foodchem.2020.127647, PMID: 32739816

[43] Burgos G Amoros W Muñoa L Sosa P Cayhualla E Sanchez C. Total phenolic, total anthocyanin and phenolic acid concentrations and antioxidant activity of purple-fleshed potatoes as affected by boiling. J Food Compos Anal. (2013) 30:6–12. doi: 10.1016/j.jfca.2012.12.001

[44] D’amelia V Sarais G Fais G Dessì D Giannini V Garramone R. Biochemical characterization and effects of cooking methods on Main phytochemicals of red and purple potato tubers, a natural functional food. Foods. (2022) 11:384. doi: 10.3390/foods11030384, PMID: 35159533 PMC8834363

[45] Managa MG Shai J Thi Phan AD Sultanbawa Y Sivakumar D. Impact of household cooking techniques on African nightshade and Chinese cabbage on phenolic compounds, antinutrients, *in vitro* antioxidant, and β-glucosidase activity. Front Nutr. (2020) 7:580550. doi: 10.3389/fnut.2020.580550, PMID: 33409289 PMC7779405

[46] Heras-Ramírez ME Quintero-Ramos A Camacho-Dávila AA Barnard J Talamás-Abbud R Torres-Muñoz JV. Effect of blanching and drying temperature on polyphenolic compound stability and antioxidant capacity of apple pomace. Food Bioprocess Technol. (2012) 5:2201–10. doi: 10.1007/s11947-011-0583-x

[47] Guzik P Kulawik P Zając M Migdał W. Microwave applications in the food industry: an overview of recent developments. Crit Rev Food Sci Nutr. (2022) 62:7989–8008. doi: 10.1080/10408398.2021.1922871, PMID: 33970698

[48] Mane S Bremner DH Tziboula-Clarke A Lemos MA. Effect of ultrasound on the extraction of total anthocyanins from purple majesty potato. Ultrason Sonochem. (2015) 27:509–14. doi: 10.1016/j.ultsonch.2015.06.021, PMID: 26186873

[49] Chaaban H Ioannou I Chebil L Slimane M Gerardin C Paris C. Effect of heat processing on thermal stability and antioxidant activity of six flavonoids. J Food Process Preserv. (2017) 41:e13203. doi: 10.1111/jfpp.13203, PMID: 36610086

[50] Severini C Giuliani R De Filippis A Derossi A De Pilli T. Influence of different blanching methods on color, ascorbic acid and phenolics content of broccoli. J Food Sci Technol. (2016) 53:501–10. doi: 10.1007/s13197-015-1878-0, PMID: 26787969 PMC4711404

[51] Desmond A Elias L Nde DB. Optimization of the blanching of potato (*Solanum tuberosum* L) slices by response surface methodology: effect on the vitamin C content and drying kinetics. Sustain Chem Eng. (2020) 17–32. doi: 10.37256/sce.11202082.17-32

[52] Xanthakis E Gogou E Taoukis P Ahrné L. Effect of microwave assisted blanching on the ascorbic acid oxidase inactivation and vitamin C degradation in frozen mangoes. Innov Food Sci Emerg Technol. (2018) 48:248–57. doi: 10.1016/j.ifset.2018.06.012

[53] Abano E. Microwave and blanching pretreatments for hot air drying of orange-fleshed sweet potato slices (*ipomoea batatas*). Int J Food Sci. (2020) 2020:1–12. doi: 10.1155/2020/8872429, PMID: 33145339 PMC7599404

[54] Abbas S Da Wei C Hayat K Xiaoming Z. Ascorbic acid: microencapsulation techniques and trends: a review. Food Rev Int. (2012) 28:343–74. doi: 10.1080/87559129.2011.635390

[55] McDougall GJ Dobson P Jordan-Mahy N. Effect of different cooking regimes on rhubarb polyphenols. Food Chem. (2010) 119:758–64. doi: 10.1016/j.foodchem.2009.07.030

[56] Orsavová J Hlaváčová I Mlček J Snopek L Mišurcová L. Contribution of phenolic compounds, ascorbic acid and vitamin E to antioxidant activity of currant (Ribes L.) and gooseberry (*Ribes uva-crispa* L.) fruits. Food Chem. (2019) 284:323–33. doi: 10.1016/j.foodchem.2019.01.072, PMID: 30744864

[57] Tena N Martin J Asuero AG. State of the art of anthocyanins: antioxidant activity, sources, bioavailability, and therapeutic effect in human health. Antioxidants. (2020) 9:451. doi: 10.3390/antiox9050451, PMID: 32456252 PMC7278599

[58] Marzuki SU Pranoto Y Khumsap T Nguyen LT. Effect of blanching pretreatment and microwave-vacuum drying on drying kinetics and physicochemical properties of purple-fleshed sweet potato. J Food Sci Technol. (2021) 58:2884–95. doi: 10.1007/s13197-020-04789-5, PMID: 34294950 PMC8249554

[59] Ali MA Yusof YA Chin NL Ibrahim MN. Effect of different drying treatments on colour quality and ascorbic acid concentration of guava fruit. Int Food Res J. (2016) 23:S155–61. Available at: https://web.s.ebscohost.com/abstract?direct=true&profile=ehost&scope=site&authtype=crawler&jrnl=19854668&AN=121329285&h=lME6ZMQWdxw8opGKa%2fnyDGUcteDiZVInyBTZqOCuqBL6SlNBq0Uy5LstHtxBEi%2bCYfk7s0up2YFR994b24Ldag%3d%3d&crl=c&resultNs=AdminWebAuth&resultLocal=ErrCrlNotAuth&crlhashurl=login.aspx%3fdirect%3dtrue%26profile%3dehost%26scope%3dsite%26authtype%3dcrawler%26jrnl%3d19854668%26AN%3d121329285

[60] Akbari N Mohammadzadeh Milani J Biparva P. Functional and conformational properties of proteolytic enzyme-modified potato protein isolate. J Sci Food Agric. (2020) 100:1320–7. doi: 10.1002/jsfa.10148, PMID: 31742702

[61] Abdullah AHD Chalimah S Primadona I Hanantyo MHG. Physical and chemical properties of corn, cassava, and potato starches. IOP Conf Earth Environ Sci. (2018) 160:012003. doi: 10.1088/1755-1315/160/1/012003

[62] Jagadeesan S Govindaraju I Mazumder N. An insight into the ultrastructural and physiochemical characterization of potato starch: a review. Am J Potato Res. (2020) 97:464–76. doi: 10.1007/s12230-020-09798-w

[63] Romano A D’Amelia V Gallo V Palomba S Carputo D Masi P. Relationships between composition, microstructure and cooking performances of six potato varieties. Food Res Int. (2018) 114:10–9. doi: 10.1016/j.foodres.2018.07.033, PMID: 30361005

[64] Gomide AI Monteiro RL Augusto B Carciofi M. The effect of Pretreatments on the physical properties and starch structure of potato chips dried by microwaves under vacuum. Foods. (2022) 11:2259. doi: 10.3390/foods11152259, PMID: 35954025 PMC9368230

[65] Sahin S Sumnu SG Sahin S Sumnu SG. Rheological properties of foods. Phys Prop foods. (2006) 39–105. doi: 10.1007/0-387-30808-3_2

[66] Jiang H Ling B Zhou X Wang S. Effects of combined radio frequency with hot water blanching on enzyme inactivation, color and texture of sweet potato. Innov Food Sci Emerg Technol. (2020) 66:102513. doi: 10.1016/j.ifset.2020.102513

[67] Tian J Chen S Wu C Chen J Du X Chen J. Effects of preparation methods on potato microstructure and digestibility: an *in vitro* study. Food Chem. (2016) 211:564–9. doi: 10.1016/j.foodchem.2016.05.112, PMID: 27283668

[68] Guo Z Zeng S Lu X Zhou M Zheng M Zheng B. Structural and physicochemical properties of lotus seed starch treated with ultra-high pressure. Food Chem. (2015) 186:223–30. doi: 10.1016/j.foodchem.2015.03.069, PMID: 25976814

[69] Leivas CL Da Costa FJOG De Almeida RR De Freitas RJS Stertz SC Schnitzler E. Structural physico-chemical, thermal and pasting properties of potato (*Solanum tuberosum* L.) flour: study of different cultivars and granulometries. J Therm Anal Calorim. (2013) 111:2211–6. doi: 10.1007/s10973-012-2395-2

[70] Chung H Liu Q. Molecular structure and physicochemical properties of potato and bean starches as affected by gamma-irradiation. Int J Biol Macromol. (2010) 47:214–22. doi: 10.1016/j.ijbiomac.2010.04.019 20438750

